# Supplementary material for: Evaluation of the feasibility and acceptability of an integrative group psychological intervention for people with Multiple Sclerosis: A study protocol
Source: PLoS One. 2023 Jul 21;18(7):e0288295. doi: 10.1371/journal.pone.0288295 (PMC10361468; doi:10.1371/journal.pone.0288295)
Supplement: S1 Protocol — (PDF) [file pone.0288295.s001.pdf]

Study Protocol

Study Title : MyMS-Ally Group Psychological Intervention: a feasibility and acceptability study of a novel group psychological intervention for people with Multiple Sclerosis

IRAS Project ID: 310410

Date and Version No: 20/09/2022; V7

Chief Investigator:

Dr Evangelia (Eva) Fragkiadaki, Psy.D.
Senior Lecturer in Counselling Psychology
Programme Leader of the Professional Doctorate Programme in Counselling Psychology
University of West of England (UWE Bristol)
Faculty of Health and Applied Sciences
Frenchay Campus, Coldharbour Lane, BS16 1QY
00447708896706
Eva.fragkiadaki@uwe.ac.uk

Investigators:

1) **Dr Nikki Cotterill**

Professor in Continence Care and Florence Nightingale Foundation Leadership Scholar 2019
Centre for Health and Clinical Research, UWE
Continence Lead, Bristol Urological Institute, NBT and BABCON
HIT Director, Bristol Health Partners
Faculty of Health and Applied Sciences, School of Health and Social Wellbeing, University of the West of England, Blackberry Hill
Bristol. BS16 1DD
T: 0117 3286863

2) **Dr Claire Rice**

Consultant Neurologist and Associate Professor in Neuroinflammation
Deputy School Education Director, Bristol Medical School
University of Bristol and North Bristol NHS Trust

Sponsor:

University of the West of England
Frenchay Campus, Coldharbour Lane, BS16 1QY

Funder: **University of the West of England**
Vice Chancellors' Early Career Researcher Award

Confidentiality Statement

This document contains confidential information that must not be disclosed to anyone other than the Sponsor, the Investigator Team, HRA, host organisation, and members of the Research Ethics Committee, unless authorised to do so.

Summary of the Study

Multiple Sclerosis (MS) is characterised by significant symptom diversity and complexity. The unpredictability of the symptoms and the emotional and cognitive facets of the disease have a significant impact on the patients' quality of life, relationships and other significant areas of living. Psychological interventions and their efficacy have been the focus of MS literature over the past years and have been found to have moderate effects on quality of life, depression and stress reduction, improvement of wellbeing, anxiety, fatigue and sleep disturbances and emotion regulation. The present research project follows from an exploratory mixed method study on the effects of various forms of psychosocial interventions on the experience and management of MS (Fragkiadaki et al., 2021). The results of that study generated themes that led to the development of a novel, patient – led psychological intervention named MyMS-Ally. Most interventions so far are based on generic models of therapy which cannot cover the complexity and unpredictability of MS. It is aspired that this patient-led model of intervention will meet the needs and preferences of people with MS. The current study aims to explore the feasibility and acceptability of MyMS-Ally intervention and also obtain preliminary data on the effects on quality of life, emotion regulation, depression and anxiety through the application of a mixed methods design. People with MS will be recruited at the Bristol and Avon Multiple Sclerosis centre. They will participate in MyMS-Ally group intervention for 8 weeks. They will complete quantitative measures before and at the end of the intervention as well as at one and three – months follow – up. Individual semi – structured qualitative interviews will also be conducted before and at the end of the intervention and at 3 – months follow-up. The aim is to explore the relevance, sustainability and adherence to the intervention and study processes (feasibility) as well as the appropriateness of the intervention based on the emotional and cognitive responses, satisfaction and perceived effectiveness (acceptability). The secondary aim of the study is to obtain preliminary data on change processes and outcomes. The study is funded by the Vice – Chancellor's Early Career Researcher Award, University of the West of England.

References

Fragkiadaki, E., Anagnostopoulos, F. & Triliva, S. (2022). Multiple Sclerosis Patients' Experience of Psychosocial Interventions: a Mixed Method study towards a Patient-Centred Approach to Intervention Development. *Sage Open (under review).*

Summary of Main Issues

Purpose and design

There are approximately 106.000 Multiple Sclerosis (MS) cases recorded in the United Kingdom (Public Health England,2020). Living with MS places the person in precarious positioning where continuous behavioural and cognitive modifications are required in confronting physical and psychosocial challenges. People with Multiple Sclerosis face the challenge of integrating the disease into their identity (Dennison, Yardley, Devereux, & Moss-Morris, 2013; Irvine, Davidson, Hoy, & Lowe-Strong, 2009) as they strive to develop a sense of coherence and control over their illness. These processes are positively related to emotional, psychological, and social wellbeing (Bassi et al., 2019; Calandri , Graziano, Borghi & Bonino, 2018). Adjustment to the illness has been associated with positive affect, optimism, self – efficacy and meaning making processes (Calandri et al., 2018). The role of psychotherapy in these processes requires further investigation (Calandri et al., 2018) through systematic exploratory analysis of diverse perspectives (Methley, Chew-Graham, Campbell & Cheraghi-Sohi, 2015).

The literature on psychological interventions has mainly focused on theory-driven models of intervention with Cognitive Behavioural Therapy (CBT) and Mindfulness being the most prominent ones. They have been associated with improvement of wellbeing (Simpson, Simpson, Ramparsad, Lawrence, Booth & Mercer, 2019; Spinzer & Pakenham, 2018), reduction of stress, anxiety, depression, fatigue and sleep disturbances (De la Torre, Mato, Doval, Espinosa et al., 2020; Hind, Cotter, Thake, Bradurn et al., 2014; Miller, Altaras, Vissicchio, Zemon, Portnoy et al., 2020; Pagnini, Cavalera, Rovaris, Mendozzi et al., 2019; Reynard, Burleson – Sullivan & Rae – Gant, 2014). More integrative approaches such as Acceptance and Commitment Therapy and supportive therapy and counselling have been found to contribute to improvements in quality of life, adjustment and coping with MS (Han, 2021; Pakenham, Mawdsley, Brown & Burton, 2018; Molton, Koelmel, Curran, Von Geldern, Ordway & Alschuler, 2019; Topcu et al., 2020).

The recent literature focuses mainly on generic models of psychological interventions which cannot cover the complexity and the unpredictability of MS. However, given recent developments of process – based psychological interventions, psychological approaches can be formulated based upon the specific characteristics of the disease for people with MS. The people with MS' voices and preferences can guide psychological intervention programmes development as well as methods of evaluation of these programmes. The current research

project aims to address the above issues, following from a longitudinal, exploratory, mixed method study on the experience of psychosocial interventions for 20 people with MS (Fragkiadaki, Anagnostopoulos & Triliva, 2021). Results of this study revealed the interplay between MS and psychosocial interventions and the journey of the participants from the despair at diagnosis, helplessness and punishment to the new preferred identity that encompasses the MS body, empowerment and forgiveness. The themes of the study guided the development of the novel group psychological intervention MyMS-Ally and the current research project explores the feasibility and acceptability of this intervention programme.

The primary objective of the current study is the investigation of the feasibility and acceptability of the novel group psychological intervention MyMS-Ally. As a secondary objective, the researchers will collect primary data to evaluate the effects of the intervention on quality of life, depression, anxiety and emotion regulation. A mixed method design will be implemented. Qualitative individual interviews will provide insight into the participants' experiences taking part in the intervention, how they perceive the impact on their experience and management of MS and which intervention processes they perceived as helpful or unhelpful. Quantitative outcome measures will record any potential change. The aim of the study is to reach conclusions with regards to the relevance and appraisals of participants taking part in the intervention so that final modifications are made and the intervention can be further evaluated. Additionally, the study aims to reach statistically significant results that will indicate the impact of the intervention but also to indicate effects that are meaningful in everyday work with people with MS (Kazdin, 1999).

Potential issues

A potential issue that may be raised in the study design is the demanding nature of the data collection methods. Participants will be asked to complete a battery of questionnaires (total 29 items) before, at the end of the intervention and at one and three – months follow - up. Participants will also be asked to engage in individual qualitative semi-structured interviews at baseline, at the end of the intervention and at 3 – months follow up. The potential burden of this level of data collection has been considered therefore short questionnaires have been selected to measure quality of life, depression, anxiety and emotion regulation. It is anticipated that it will take participants 10 – 15 minutes to complete the questionnaires. Online administration has been suggested in the study protocol. Moreover, the interviews have been planned to be conducted online through Microsoft Teams. The interviews will respect the participants' pace, needs and preferences; for example, have regular breaks during the interview. Moreover, following PPI involvement input, flexibility will be implemented in data collection stage so participants might prefer oral administration of questionnaires and/or they might prefer they have their carers attending the data collection sessions. It will be ensured that all potential participants will be fully informed of the study procedures prior to giving their consent.

The present research project has been discussed and reviewed by two experienced researchers in the field of Multiple Sclerosis and health research. Dr Nikki Cotterill, Associate Professor at the University of the West of England is the internal to the sponsor project supervisor. Dr Claire Rice, Consultant Neurologist and Associate Professor in University of Bristol and North Bristol NHS Trust is overseeing the study procedures at the Bristol and Avon Multiple Sclerosis Centre where the recruitment and implementation of the intervention will take place. Moreover, the Research Network of the UK MS Society has been involved, consulting on the intervention implementation, the study design as well as dissemination strategies. Their input has affected the intervention implementation and study processes enhancing our aim to minimise risk and burden for participants and researchers.

Recruitment and Inclusion/ Exclusion Criteria

Participants will be recruited at the Bristol and Avon Multiple Sclerosis Centre, Southmead Hospital, North Bristol NHS Trust. The study will be presented to the multidisciplinary team of professionals who will then disseminate the research information, participant information sheet and consent form to people with Multiple Sclerosis (MS) that fit the inclusion and exclusion criteria. The potential participants who will be interested in participating or finding out more about the project will contact the Chief Investigator (CI) via phone, text or email (according to their preference) and they will have an initial discussion on what the intervention and study entail. This discussion will also serve as the initial screening where the CI will be able to explore how the potential participant fits the inclusion and exclusion criteria:

Inclusion Criteria

- Participants must be able to read, write and speak English.
- Participants must be willing and able to give informed consent for participation in the study.
- Participants can be people who identify as men, women, non – binary, aged 18 and above.
- Participants must be diagnosed with Multiple Sclerosis and have received their first diagnosis more than two years ago.
- Participants must be cognitively and physically ready and willing to engage into eight weekly group psychological intervention sessions (discussed in screening meeting with potential participant).

- Participants should have access to the internet and to a device that will allow them to join the intervention and data collection processes through Microsoft Teams programme.

Exclusion Criteria
The participants may not enter the study if ANY of the following apply:

- People with MS with suspected or diagnosed comorbidity with Depression, Bipolar Disorder or Psychotic Disorders (discussed at the screening stage with CI).
- People with MS that have received the MS diagnosis in the last two years prior to data collection point (avoid effects of newly diagnosed people with MS who would still be contemplating and making therapy decisions, there are specific needs that characterise this population).
- People with MS who are attending another psychotherapy or psychological intervention process at the time of application of MyMS-Ally group intervention.

All potential participants who will express their interest in the study will contact the CI and have an initial screening meeting and conversation. No one will be unfairly excluded from or included in the research. There will be no exceptions made regarding eligibility, i.e., that each participant must satisfy all the approved inclusion and exclusion criteria of the protocol. Potential participants will be aware of the intervention implementation design and the study procedures before they make an informed decision to participate in the study. The CI will be available to answer any further questions and provide clarifications after the initial/ screening discussion until the people with MS make their final decision. The potential participants will have two weeks to decide whether they are willing to participate after the initial meeting with the CI. The CI is a Chartered Counselling Psychologist with many years of clinical and academic experience and has been working with people with MS in a research context for the last five years. She feels confident she has the capacity to discuss and assess the impact the study will have on potential participants and also comprehends the ethical principles of delivering and evaluating a psychological intervention.

Those who will agree to participate will be asked to provide the CI with their demographics, email and telephone contact details in order to commence with the data collection process as well as to inform them of the start of the group intervention. Only the CI will have access to the participants' personal details. Five to six participants will be recruited and allocated to one group which will last two months (eight weekly sessions) following PPI involvement input. If more people with MS show interest in participating in the study, then more that one groups will be offered.

Consent

The participants must personally sign and date the latest approved version of the Informed Consent form before any study specific procedures are performed.

Written versions of the Participant Information Sheet and Informed Consent will be presented to the participants detailing the exact purpose and nature of the study, what it will involve for the participant, the known possible risks and benefits involved in taking part. It will be clearly stated that the participant is free to withdraw from the study for any reason without prejudice to future care, without affecting their legal rights, and with no obligation to give the reason for withdrawal.

The participants will have this information long enough to consider before making an informed decision. The maximum timeframe is two weeks after the initial screening meeting with the CI. Participants will also have the opportunity to question the CI or other independent parties to decide whether they will participate in the study. Written Informed Consent will then be obtained by means of participant dated signature and dated signature of the CI who will present and obtain the Informed Consent. Potential participants might express interest when the recruitment stage of the study will be completed. They will still have the opportunity to have an initial/ screening discussion with CI and with their consent their contact details will be retained by the CI for future research opportunities with their consent. A copy of the signed Informed Consent will be given to the participant. The original signed form will be retained at the study site. The health professional who referred the participant to the study will be informed verbally by the CI that their service user is taking part in the study.

Risks, burdens and benefits

No serious adverse events are expected as a result of the study design and procedures. However, incidents might take place during the course of the study and the implementation of the psychological intervention:

- Participants might find the participation in a group intervention distressing and tiring.
- Participants might find the quantitative data collection process burdensome.
- Participants might not be able to attend the individual qualitative interviews.
- The group facilitator and the supervisor might make the decision that one or more participants cannot engage into the group intervention with the rest of the members.

The intervention and the study processes have been designed in a way that they respect the participants' needs and characteristics. The research team will follow the participants' pace and will take under consideration factors such as cognitive and physical fatigue in the intervention implementation and data collection processes. Following the PPI involvement input, the intervention and data collection processes will take place online and will be implemented in a flexible way to accommodate participants' needs. Therefore, should any participant miss a session during the intervention implementation because of health issues they will still have the opportunity to continue taking part in the intervention and the study.

Should any of the above adverse events occur, the participants have the right to withdraw from the study without having to justify their decision. If a participant withdraws, the CI will arrange for a check – in meeting with them if they accept the invitation and support resources will be provided to all participants in the Participant Information Sheet. Moreover, all data from participants that withdraw from the study will not be used in the analysis and will be destroyed along with any personal details.

The benefits for participants entail:

- It is aspired that participants will experience the benefits of the intervention in their experience and management of MS, their quality of life and emotion regulation.
- Taking part in a novel group psychological intervention which has been developed by people with MS' accounts of needs and preferences, psychotherapy theory and consultation with practitioners.
- Contributing to ameliorating and finalising the intervention protocol which will be disseminated to patients and practitioners involved in the care of people with MS.
- Participants voices will be heard in terms of how they experience their MS and the psychological intervention through the qualitative processes of collection of data which will provide significant insights into the complexity of the disease and the effects of the intervention as well as future research in the field.

The research study and intervention have been developed based on a patient – centred approach and all procedures have been designed in a way that they protect the participants from the above adverse events and facilitate the benefits for them. Following PPI involvement input, no major amendments were suggested and the members agreed with the suggested risks and benefits. Their recommendation was to ensure that the benefit of participants experiencing the benefits of the intervention in their experience and management of MS should be first in the list.

Confidentiality

The minimum necessary person – identifiable information will be collected for the purposes of the study. The study will comply with the General Data Protection Regulation (GDPR) and Data Protection Act 2018, which require data to be de-identified as soon as it is practical to do so. The processing of the personal data of participants will be minimised by making use of a unique participant study pseudonym on all study documents and any electronic database. All documents will be stored securely on the University of the West of England One Drive in password – protected folders and will only be accessible by study staff. The CI will safeguard the privacy of participants' personal data.

The personal details that will be collected from the participants will include name, surname, email, phone number so that the CI will have a way to contact participants for the

intervention implementation and the collection of data arrangements. No other members of the research team will have access to participants' personal details. In the event that the CI and the group intervention facilitator assess that participants or others are at serious risk then the care professional who referred them to the study will be contacted to discuss further steps.

Conflict of interest

The roles of each member of the research team are clearly defined. The CI is the lead researcher of the study and is not involved in the implementation of the group psychological intervention MyMS-Ally or the collection of the qualitative data. The group facilitator will be a trained practitioner who will be supervised by a senior practitioner and they will comprise the clinical team of the study who will not be involved in the collection and analysis of the data. The qualitative interviews will be conducted by an experienced independent researcher. Therefore, research and clinical processes are clearly separated in the course of the study. The CI's identity as a Chartered Counselling Psychologist and her clinical skills will be implemented in order to create safe and respectful environments for participants in the processes of intervention implementation and collection of data.

At the end of the study participants will have the opportunity to meet with the CI for check – in meetings should they accept the invitation. The final results of the study will also be fed back to the participants should they accept the report. Data will be stored in the University of the West of England Research Repository and will be reserved there as restricted data accessed by third parties for purposes of review. The participants' personal details will be destroyed according to guidelines.

References

Bassi, M., Grobberio, M., Negri, L., Cilia, S., Minacapelli, E., Niccolai, C., Pattini, M., Pietrolongo, E., Quartuccio, M. E., Viterbo, R. G., Allegri, B., Amato, M. P., Benin, M., De Luca, G., Falautano, M., Gasperini, C., Patti, F., Trojano, M., & Delle Fave, A. (2019). The Contribution of Illness Beliefs, Coping Strategies, and Social Support to Perceived Physical Health and Fatigue in Multiple Sclerosis. *Journal of Clinical Psychology in Medical Settings, 28*(1), 149–160. https://doi.org/10.1007/s10880-019-09692-6

Calandri, E., Graziano, F., Borghi, M., & Bonino, S. (2018). Depression, Positive and Negative Affect, Optimism and Health-Related Quality of Life in Recently Diagnosed Multiple Sclerosis Patients: The Role of Identity, Sense of Coherence, and Self-efficacy. *Journal of Happiness Studies*, *19*(1), 277–295. https://doi.org/10.1007/s10902-016-9818-x

De la Torre, G. G., Mato, I., Doval, S., Espinosa, R., Moya, M., Cantero, R., Gonzalez, M., Gonzalez, C., Garcia, M. A., Hermans, G., González-Torre, S., Mestre, J. M., & Hidalgo, V. (2020). Neurocognitive and emotional status after one-year of mindfulness-based intervention in patients with relapsing-remitting multiple sclerosis. *Applied Neuropsychology:Adult*, *0*(0), 1–10. https://doi.org/10.1080/23279095.2020.1732388

Dennison, L., Moss-morris, R., Yardley, L., Kirby, S. & Chalder, T. (2013). Change and processes of change within interventions to promote adjustment to multiple sclerosis : Learning from patient experiences. *Psychology & Health*, 28(9), 973 - 992. https://doi.org/10.1080/08870446.2013.767904

Fragkiadaki, E., Anagnostopoulos, F. & Triliva, S. (2021). Multiple Sclerosis Patients' Experience of Psychosocial Interventions: a Mixed Method study towards a Patient-Centred Approach to Intervention Development. *Disability and Rehabilitation (under review).*

Han, A. (2021). Effects of mindfulness-and acceptance-based interventions on quality of life , coping , cognition , and mindfulness of people with multiple sclerosis : a systematic review and meta-analysis. *Psychology, Health & Medicine*, *00*(00), 1–18. https://doi.org/10.1080/13548506.2021.1894345

Hind, D., Cotter, J., Thake, A., Bradburn, M., Cooper, C., Isaac, C., & House, A. (2014). Cognitive behavioural therapy for the treatment of depression in people with multiple sclerosis: A systematic review and meta-analysis. *BMC Psychiatry*, *14*(1). https://doi.org/10.1186/1471-244X-14-5

Irvine, H., Davidson, C., Hoy, K., & Lowe-Strong, A. (2009). Psychosocial adjustment to multiple sclerosis: exploration of identity redefinition. *Disability and rehabilitation*, *31*(8), 599-606.

Kazdin, A. E. (1999). The meanings and measurement of clinical significance. *Journal of Consulting and Clinical Psychology*, *67*(3), 332–339. https://doi.org/10.1037/0022-006X.67.3.332

Methley, A. M., Chew-Graham, C., Campbell, S., & Cheraghi-Sohi, S. (2015). Experiences of UK health-care services for people with multiple sclerosis: A systematic narrative review. Health Expectations, 18(6), 1844–1855. https://doi.org/10.1111/hex.12228

Miller, J. R., Altaras, C., Vissicchio, N. A., Zemon, V., Portnoy, J. G., Gromisch, E. S., Sloan, J., Tyry, T., & Foley, F. W. (2020). The influence of trait mindfulness on depression in multiple sclerosis: potential implications for treatment. *Quality of Life Research*, *29*(12), 3243–3250. https://doi.org/10.1007/s11136-020-02567-6

Molton, I. R., Koelmel, E., Curran, M., Von Geldern, G., Ordway, A., & Alschuler, K. N. (2019). Pilot intervention to promote tolerance for uncertainty in early multiple

sclerosis. *Rehabilitation Psychology*, *64*(3), 339–350. https://doi.org/10.1037/rep0000275

Pagnini, F., Cavalera, C., Rovaris, M., Mendozzi, L., Molinari, E., Phillips, D., & Langer, E. (2019). Longitudinal associations between mindfulness and well-being in people with multiple sclerosis. *International Journal of Clinical and Health Psychology*, *19*(1), 22–30. https://doi.org/10.1016/j.ijchp.2018.11.003

Pakenham, K. I., Mawdsley, M., Brown, F. L., & Burton, N. W. (2018). Pilot evaluation of a resilience training program for people with multiple sclerosis. *Rehabilitation Psychology*, *63*(1), 29–42. https://doi.org/10.1037/rep0000167

Public Health England [2020]. Multiple Sclerosis: Prevalence, Incidence and Smoking Status—Data Briefing

Reynard, A. K., Sullivan, A. B., & Rae-Grant, A. (2014). A systematic review of stress-management interventions for multiple sclerosis patients. *International Journal of MS Care*, *16*(3), 140–144. https://doi.org/10.7224/1537-2073.2013-034

Simpson, R., Simpson, S., Ramparsad, N., Lawrence, M., Booth, J., & Mercer, S. W. (2019). Mindfulness-based interventions for mental well-being among people with multiple sclerosis: A systematic review and meta-analysis of randomised controlled trials. *Journal of Neurology, Neurosurgery and Psychiatry*, *90*(9), 1051–1058. https://doi.org/10.1136/jnnp-2018-320165

Spitzer, E., & Pakenham, K. I. (2018). Evaluation of a brief community-based mindfulness intervention for people with multiple sclerosis: A pilot study. *Clinical Psychologist*, *22*(2), 182–191. https://doi.org/10.1111/cp.12108

Topcu, G., Griffiths, H., Bale, C., Trigg, E., Clarke, S., Potter, K. J., Mhizha-Murira, J. R., Drummond, A., Evangelou, N., Fitzsimmons, D., & das Nair, R. (2020). Psychosocial adjustment to multiple sclerosis diagnosis: A meta-review of systematic reviews. *Clinical Psychology Review*, *82*(September), 101923. https://doi.org/10.1016/j.cpr.2020.101923

Principal question/ objective

The study aims to explore the feasibility and acceptability of the novel group psychological intervention MyMS-Ally. This is a mixed method study and collection of data aims to provide insight on relevance, sustainability and adherence to the intervention and study processes (feasibility) as well as the appropriateness of the intervention based on the emotional and cognitive responses, satisfaction and perceived effectiveness (acceptability) based on participants' accounts and experience of the intervention. The findings are aspired

to help make the appropriate modifications before the intervention is further evaluated in future research (Bowen et al., 2009).

References

Bowen, D., Kreuter, M., Spring, B., Cofta-Woerpel, L., Linnan, L., & Weiner, D. et al. (2009). How We Design Feasibility Studies. *American Journal Of Preventive Medicine*, *36*(5), 452-457. doi: 10.1016/j.amepre.2009.02.002

Secondary objectives

The study aims to collect preliminary data on the impact of the intervention on the participants' quality of life, depression, anxiety and emotion regulation. Outcome measures will be administered at baseline, on completion of the intervention, one month and three months follow – up. Change will be recorded in the outcome measures. Participants' perceived change on the experience and management of MS associated to intervention processes will also be explored in the qualitative interviews. Clients' perspectives offer a significant insight on the impact of psychological intervention processes and effectiveness (Fragkiadaki et al., 2021).

References:

Fragkiadaki, E., Triliva, S. & Anagnostopoulos, F. (2021). Application of Interpretative Phenomenological Analysis methodology in psychotherapy impact research: Experience of psychotherapy of a person with Multiple Sclerosis. *Qualitative Methods in Psychology Bulletin, 31,* 26 – 37.

Scientific justification of the research

Literature has focused extensively on the psychiatric and psychological dimensions of Multiple Sclerosis (MS) (Gay et al., 2017). People with MS have a higher prevalence of anxiety and depression than the general population (Marie et al., 2017). Developing a sense of coherence and control over their illness has been positively associated with people with MS' emotional, psychological and social wellbeing (Bassi et al., 2016; Calandri et al., 2018). The role of psychological interventions in these processes has been explored in previous research and the results indicate how interventions such as Cognitive Behavioural Therapy, Mindfulness and Acceptance and Commitment Therapy have moderate effects on fatigue, pain, resilience, positive affect, anxiety, depression and stress management (Alschler et al., 2020; Moss – Morris et al., 2021; Pakenham et al., 2018; Taylor et al., 2020).

Previous research is mainly based on quantitative outcome measures to capture change processes as a result of the interventions. The findings present the effects of these generic models on psychological and physical facets of MS. However, little is known about the experience of people with MS that take part in these interventions and especially about their needs and preferences with regards to psychological programmes involved in their care. The current study follows from a mixed method longitudinal exploratory study on the experience of psychosocial interventions for 20 people with MS (Fragkiadaki et al., 2022). The findings of the study revealed the trajectories of people with MS from diagnosis through their engagement into individual and group psychological interventions of their choice in the community. Forgiveness, empowerment and familiarisation with the new body contributed to feelings of relief with a significant impact on improvement of physical functioning, perception of general health and reduction of anxiety (Fragkiadaki et al., 2022).

The novel intervention MyMS-Ally developed for the current project adopted a process – based approach (Hofmann & Hayes, 2019). Intervention techniques and processes were informed by aspects of established therapeutic approaches as they have been found effective for people with MS based on previous research conclusions. In addition to this, the goal of the current research project is to move away from theory – based interventions and towards a patient – led perspective of intervention development and implementation. We followed the trend in recent literature on clinical therapy processes to move from nomothetic to idiographic approaches of research and intervention (Hofmann & Hayes, 2019). As we have been developing MyMS-Ally group psychological intervention, our aspiration has been to establish processes that correspond to procedures, needs and characteristics of people with MS (Hofmann & Hayes, 2019). Thus, the structure of this intervention has also been based on what people with MS have found significant, helpful and beneficial in their accounts of their experience of psychological interventions they have engaged into (Fragkiadaki et al., 2022). The themes represented the change processes from the people with MS' perspective as they related them to intervention processes. These themes guided the development of the novel psychological group intervention MyMS-Ally.

Following the systematic development of the intervention, it needs to be tested carefully through pilot feasibility and acceptability studies in order to address uncertainties and make appropriate modifications before further evaluation of the intervention (Medical Research Council Guidance). The current study will provide valuable information on how the intervention will be appraised by participants that take part, on helpful and unhelpful aspects of the intervention based on the participants' experiences and accounts as well as the feasibility of implementation of the intervention in the context of the service. Moreover, adherence to outcome measures and study processes will also be assessed which will give valuable information for the design of the consequent evaluation study (Craig et al., 2008).

References

Alschuler, K. N., Altman, J. K., & Ehde, D. M. (2020). Feasibility and Acceptability of a Single-Session, Videoconference-Delivered Group Intervention for Pain in Multiple Sclerosis. *Rehabilitation Psychology*, *66*(1), 22–30.

Bassi, M., Grobberio, M., Negri, L., Cilia, S., Minacapelli, E., Niccolai, C., Pattini, M., Pietrolongo, E., Quartuccio, M. E., Viterbo, R. G., Allegri, B., Amato, M. P., Benin, M., De Luca, G., Falautano, M., Gasperini, C., Patti, F., Trojano, M., & Delle Fave, A. (2019). The Contribution of Illness Beliefs, Coping Strategies, and Social Support to Perceived Physical Health and Fatigue in Multiple Sclerosis. *Journal of Clinical Psychology in Medical Settings*, *28*(1), 149–160. https://doi.org/10.1007/s10880-019-09692-6

Calandri, E., Graziano, F., Borghi, M., & Bonino, S. (2018). Depression, Positive and Negative Affect, Optimism and Health-Related Quality of Life in Recently Diagnosed Multiple Sclerosis Patients: The Role of Identity, Sense of Coherence, and Self-efficacy. *Journal of Happiness Studies*, *19*(1), 277–295. https://doi.org/10.1007/s10902-016-9818-x

Craig, P., Dieppe, P., Macintyre, S., Michie, S., Nazareth, I., & Petticrew, M. (2013). Developing and evaluating complex interventions: The new Medical Research Council guidance. *International Journal of Nursing Studies*, *50*(5), 587–592. https://doi.org/10.1016/j.ijnurstu.2012.09.010

Fragkiadaki, E., Anagnostopoulos, F. & Triliva, S. (2022). Multiple Sclerosis Patients' Experience of Psychosocial Interventions: a Mixed Method study towards a Patient-Centred Approach to Intervention Development. *Sage Open (under review).*

Gay, M. C., Bungener, C., Thomas, S., Vrignaud, P., Thomas, P. W., Baker, R., ... & Montreuil, M. (2017). Anxiety, emotional processing and depression in people with multiple sclerosis. *BMC neurology*, *17*(1), 1-10.

Hofmann, S. G., & Hayes, S. C. (2019). The Future of Intervention Science: Process-Based Therapy. *Clinical Psychological Science*, *7*(1), 37–50. https://doi.org/10.1177/2167702618772296

Marrie, R. A., Walld, R., Bolton, J. M., Sareen, J., Walker, J. R., Patten, S. B., Singer, A., Lix, L. M., Hitchon, C. A., El-Gabalawy, R., Katz, A., Fisk, J. D., & Bernstein, C. N. (2017). Estimating annual prevalence of depression and anxiety disorder in multiple sclerosis using administrative data. *BMC Research Notes*, *10*(1), 1–6. https://doi.org/10.1186/s13104-017-2958-1

Moss-Morris, R., Harrison, A. M., Safari, R., Norton, S., van der Linden, M. L., Picariello, F., Thomas, S., White, C., & Mercer, T. (2021). Which behavioural and exercise interventions targeting fatigue show the most promise in multiple sclerosis? A systematic review with narrative synthesis and meta-analysis. *Behaviour Research and Therapy*, *137*(June 2019), 103464. https://doi.org/10.1016/j.brat.2019.103464

Pakenham, K. I., Mawdsley, M., Brown, F. L., & Burton, N. W. (2018). Pilot evaluation of a resilience training program for people with multiple sclerosis. *Rehabilitation Psychology*, *63*(1), 29–42. https://doi.org/10.1037/rep0000167

Taylor, P., Dorstyn, D. S., & Prior, E. (2020). Stress management interventions for multiple sclerosis: A meta-analysis of randomized controlled trials. *Journal of Health Psychology*, *25*(2), 266–279. https://doi.org/10.1177/1359105319860185

Research design and methodology

This is a mixed – method pilot design with the aim to explore feasibility and acceptability of the novel group psychological intervention MyMS-Ally. Quantitative and qualitative data will be collected in order to facilitate analysis of people with MS' experience of the intervention as well as analysis of outcomes on quality of life, anxiety, depression and emotion regulation. The mixed-method design offers the framework for statistically significant conclusions to be made but also incorporate participants' meaning – making accounts with an in-depth description of their experience of the intervention. The study adheres to an idiographic paradigm of exploring the impact of the intervention (Haynes et al., 2009; Molenaar, 2004) where each participant's variations and changes over time are investigated through multiple points of collecting data. Following the patient – centred regime of the development of the intervention and the evaluation processes, the aim of the researchers is to identify tailored assessment processes that will eventually shed light on changes over time within each participant. Quantitative and qualitative methods are "compatible partners" in this research context (Onghema et al., 2019, p. 463) following a pragmatism epistemology combining action and reflection (Biesta 2010).

Participants

Recruitment and implementation of the intervention will take place in the Bristol and Avon Multiple Sclerosis Centre in Southmead Hospital, Bristol. A group of 5 - 6 participants will be recruited to take part in the MyMS-Ally group intervention. The group size reflects the minimum number of members of a group psychological intervention for meaningful interactions to be unfolded and the maximum number which allows enough time and space for each member to use the group effectively (Saracutu et al., 2018). The size of the sample also reflect the recommendations made in the PPI involvement focus group. Should more people with MS express interest in taking part, more than one groups will be offered. Eligible participants will be aged 18 years or above, diagnosed with multiple sclerosis more than two years ago, able to read, write and speak English and able to engage into eight weekly group psychological intervention sessions (2 months). The group intervention as well as data collection processes will take place online therefore access to the internet and to a device where they can use Microsoft Teams software on are required. People with MS with suspected and/or diagnosed comorbidity with Depression, Bipolar Disorder or Psychotic Disorders will not be able to participate in the study.

Recruitment will be carried out in collaboration with the team of professionals of the Bristol and Avon Multiple Sclerosis (BAMS) Centre in Southmead Hospital, Bristol. The study as well as the inclusion and exclusion criteria of the sampling will be presented to the team in a meeting. An information package will also be given to the team of professionals with relevant information on the intervention, study processes and participant eligibility. The professionals will identify patients that meet the eligibility criteria and inform them of the study. The potential participants will be given the Participant Information Sheet and the Consent Form at that point as they reflect on whether or not they are interested in taking part. People with MS who are interested in participating will contact the chief investigator (CI) who will arrange individual meetings with them to discuss the study procedures and the intervention process, the eligibility criteria as well as answer any questions they might have. The people with MS who will agree to participate will have to return the signed consent form to the CI before the commencement of collection of data and intervention.

MyMS-Ally group intervention

The intervention has been developed based on previous research conclusions, systematic reviews and meta – analyses about what is helpful for people with MS in the field of psychological intervention programmes. Moreover, following the patient – centred paradigm, the intervention is based on people with MS' accounts on change processes in their experience and management of MS in relation to intervention processes. MyMS-Ally group intervention will last for two months and participants will be asked to attend weekly sessions

that will last 1 ½ hours each (8 sessions). The details of the intervention are outlined in the attached document in this IRAS application. The intervention will take place online following the PPI involvement group recommendation. Following the PPI involvement focus group discussion, it will be ensured that the pace and the needs of the participants will be taken under account during the implementation of the intervention. For example, the facilitator will establish breaks during the sessions for the participants to take time and move away from their monitors as well as incorporate overviews and summaries of previous sessions in the discussions in the group.

Data collection

Feasibility and acceptability of the processes of the novel group intervention MyMS-Ally will be explored mainly through the qualitative interviews. The participants will attend three qualitative semi-structured interviews:

1. At baseline, before commencement of the intervention: This interview will focus on the experience of their Multiple Sclerosis and the expectations they have from MyMS-Ally group intervention (prospective acceptability, Sekhon et al., 2017).
2. At the end of the intervention (at the end of the 8 sessions): This interview will focus on satisfaction, on helpful and unhelpful aspects of the intervention as they experienced them, what facilitated them to complete the programme and any impact they perceived the intervention had on their experience and management of their Multiple Sclerosis (Sekhon et al., 2017).
3. At three months follow – up: This interview will focus on their experience of MyMS-Ally intervention and the impact it might have on their experience and management of their Multiple Sclerosis.

Interviews are expected to last approximately one hour and they will take place online on a platform with encryption where recordings are possible (Microsoft Teams) and stored on the CI’s university (UWE) onedrive in a password protected folder.

Additional indicators will be recorded in order to explore further the feasibility and acceptability of MyMS-Ally intervention (Saracutu et al., 2018):

- Number of people with MS referred by the health and mental health professionals of the service.
- Number of people with MS attending the screening meetings with the Chief Investigator.
- Time taken to complete the questionnaires and missing data.
- Follow up responses (1 month and 3 months follow up).
- Number of sessions attended by the participants.
- Feedback from MyMS-Ally group intervention facilitator.

Participants will be asked to complete three questionnaires for the collection of quantitative data and to address the secondary objectives of the study. The aim is to collect preliminary data on outcomes and impact of the intervention on quality of life, anxiety, depression and emotion regulation. Quality of life, anxiety and depression have been extensively used as indicators for change in the literature of evaluating psychological interventions for people with MS. Emotion regulation is a factor that needs to be explored further as it has been associated with the wellbeing of people with autoimmune diseases (Karademas et al., 2020). The questionnaires participants will be asked to complete are:

- Satisfaction with Life Scale (SWLS): The satisfaction with life scale was developed to assess the participants' satisfaction with life as a whole (Diener et al., 1985). It comprises of five items. It has shown sufficient sensitivity to detect change in life satisfaction during the course of a clinical intervention (Pavot & Diener, 1993) and in the field of health psychology it has been used to evaluate subjective quality of life (Pavot & Diener, 2008). The items are global rather than specific in nature. Statements are rated on a 7-point Likert scale (1 strongly disagree, 7 strongly agree).
- Hospital Anxiety and Depression Scale (HADS): The HADS is a self – report scale assessing the states of depression and anxiety. Participants are asked to reflect on how they have been feeling during the past week. The scale comprises of 14 items, seven for anxiety and seven for depression. The scale has demonstrated good factor structure and internal consistency (Mykletun et al., 2013). The scale has also been used in research with people with MS (Nazari et al., 2020).
- Emotion Regulation Questionnaire (ERQ): A 10-item scale designed to measure respondents' tendency to regulate their emotions in two ways: (1) Cognitive Reappraisal and (2) Expressive Suppression. Respondents answer each item on a 7-point Likert-type scale ranging from 1 (strongly disagree) to 7 (strongly agree). Higher scores in the reappraisal scale indicate better ability to monitor, evaluate, and reduce distress. In contrast, higher scores in the suppression scale indicate poorer ability to repair mood and to manage stressful situations. Overall, the ERQ has very good psychometric properties with a definite two-factor structure, good internal consistency (0.83 and 0.79 for reappraisal and suppression, respectively), and satisfactory test–retest reliability (Gross & John, 2003). Cognitive reappraisal has been associated with wellbeing for people with Multiple Sclerosis (Karademas et al., 2018) and has been used to assess psychological states and quality of life for people with MS (Phillips et al., 2009; Sadeghi et al., 2021).

A significant advantage of the questionnaires chosen is that they are brief (it takes about 10 – 15 minutes to complete). Participants will be asked to complete the questionnaires four times during the course of the study:

1. At baseline, before commencement of the intervention
2. At the end of the intervention
3. At one month follow up after the end of the intervention
4. At three months follow up after the end of the intervention

Methodological integrity

The study adheres to ethical guidelines as outlined by the University of West of England as well as the British Psychological Society. The CI is a Chartered Counselling Psychologist with many years of clinical and research experience in the field of mental health. She has been working with people with MS in a research context systematically since 2017. She has been interviewing participants adhering to qualitative methodology and principles and has been the main administrator of quantitative questionnaires. She is thus competent and experienced to design and conduct the current mixed method pilot project. She has been keeping a research journal where all processes are outlined in order to achieve transparency in the final report. The study processes have been reviewed by the supervisor and collaborators of the project and it has been thoroughly discussed in the PPI group where no major amendments were recommended. The analysis of the data will be discussed in the group of researchers and an experienced qualitative researcher external to the project will audit the analysis of the qualitative data to ensure integrity and trustworthiness.

Stages of research/ Timetable

Preparation and discussion in PPI group<br><br>February 2022	The PPI group has been formed through the Research Network of the UK MS Society
Finalise and submit IRAS application and FREC ethics application<br><br>February - March 2022	The final IRAS application will be submitted as soon as final approvals are received in the research team.<br><br>Following IRAS approval, all forms and approval letter will be forwarded to UWE Faculty Research Ethics Committee.

Meeting with the team of professionals at the Bristol and Avon Multiple Sclerosis Centre<br><br>Initiating recruitment stage<br><br>October 2022	The study processes and interventions will be presented to the team of professionals at BAMS centre and initial meetings with potential participants will take place.
Baseline collection of data and commencement of intervention<br><br>–November 2022	The baseline collection of quantitative and qualitative data will take place before commencement of the intervention.
Implementation of intervention<br><br>November 2022 – January 2023	Intervention will be implemented for 8 weeks after commencement. Participants will attend 8 weekly sessions which will last 1 ½ hours each.
End of intervention collection of data.<br><br>February 2023	The end of intervention collection of quantitative and qualitative data will take place
One month follow up collection of quantitative data<br><br>March 2023	One – month follow up collection of quantitative data will take place.
Three – month follow up collection of data<br><br>June 2023	Three – month follow up collection of quantitative and qualitative data will take place.
Data analysis and preliminary findings	Qualitative and quantitative data will be analysed in this period

July – September 2023	
Writing up of internal report for VC – ECR Award (funder) and publications<br><br>September 2023	Final report and articles for publication will be written.
Dissemination of findings<br><br>August – December 2023	In the same period dissemination of findings and dissemination strategies will be implemented

End of the study

The study will end when the three – month follow – up data will be collected by all participants. These will include the questionnaires completion and the qualitative interviews with the lead researcher. This is estimated to take place in June 2023.

Patient involvement statement

PPI involvement in the design

Five members of the Research Network of UK MS Society agreed and participated in a focus group with the project Chief Investigator (CI) (Eva Fragkiadaki) to discuss intervention implementation and research design. They were sent a brief overview of the research project and MyMS-Ally intervention structure. The CI also presented the intervention and the study processes briefly in the beginning of the group before moving on to the discussion. The focus group was semi – structured with specific questions that had been developed by the CI and focused on: a. intervention, b. research processes, c. dissemination. The CI also encouraged open discussion on any feedback or input members might have had on the intervention and research plan. The members' feedback is summarised below and incorporated in the intervention planning and research design:

1. PPI group members provided substantial feedback on intervention structure and delivery:
    - The intervention sessions should be timed so participants are aware how much time will be spent on each theme in the session. This is to ensure that there will be enough time without overburdening the participants.
    - They agreed that the intervention should be conducted online as it is easier for people with MS with complex needs to participate from the comfort of their

homes. It is also easier for them as they will not have to travel and it makes it easier to concentrate and tackle issues of fatigue.
    - Intervention (and data collection processes) should be scheduled for a time in the day when it's most appropriate for people with MS, therefore avoid late afternoon and evening.
    - There needs to be flexibility with attendance; even though we want participants to be committed to the intervention we should also respect issues of fatigue that might come up or "bad days" and allow people to continue to participate even if they miss a session.
    - There needs be "feedback" in the form or reminders or overview of the sessions at the end, between or in the beginning of the next session where participants will be able to remind themselves of the main themes and incidents discussed in the sessions. This will accommodate any cognitive difficulties they might experience.
2. PPI group members stated that the initial plan for 6 – 12 participants for one group will not be effective as more than 5 or 6 members in a group makes it very difficult to interact and have in – depth interactions. They strongly suggested recruiting 5 – 6 participants and if more people expressed interest in taking part then more than one groups will be offered.
3. PPI group members agreed with the sampling inclusion and exclusion criteria and highlighted areas that the research team must be mindful of as they recruit participants:
    - They suggested we do not include an upper age limit as people who are 70 years old might be more than capable and willing to share in a group which will be beneficial for the rest of the participants. Therefore, the research team changed the criterion to people above 18 without an upper age limit.
    - Researchers should consider the impact of age of participants in the group intervention.
    - Researchers should consider the impact of physical disability in the group intervention.
    - PPI group agreed that the intervention should be conducted online and therefore participants should have access to a device (computer, laptop, tablet, phone) and to the platform intervention and research data collections processes will take place on (Microsoft Teams).
4. There needs to be flexibility with data collection processes. There need to be breaks, time for rest at the time of interviews, perhaps stop an interview and go back to the participant when they are ready to continue. Moreover, there needs to be flexibility with completing questionnaires so there might be a need for the CI to administer the questionnaires verbally, online or over the phone.
5. If a participant has difficulty articulating themselves or completing questionnaires, it is sometimes useful to have a carer with them to help so this will be allowed in data collection processes.

6. In online delivery of the intervention and data collection, captions must be on in Microsoft Teams.
7. The members suggested the following organisations along with the NHS that would benefit to be involved in the dissemination of the study findings and the intervention:
    - UK MS Society
    - MS Trust
    - Shift MS
    - Overcoming MS

    They also suggested podcasts as an efficient dissemination strategy. All the above will be implemented in the dissemination of the research conclusions.

8. No major amendments were suggested in the risk and benefits part of the design. The members agreed with the suggested risks and benefits. Their recommendation was to ensure that the benefit of participants experiencing the benefits of the intervention in their experience and management of MS should be first in the list.
9. For future steps of this project, the members suggested that the research team should enhance inclusion by recruiting from organisations across the country (given that the intervention and evaluation study processes will be implemented online) as well as receiving funding to incorporate means and software that will help more people with MS with complex needs and physical difficulties to take part in the intervention and future evaluation studies.
10. Three of the five members of the PPI focus group requested to stay updated and involved in the project. Following their request, the CI will arrange group meetings with them at the important milestones of the research project and their involvement will be further discussed.

References

Biesta, G. J. J. (2010). Pragmatism and the philosophical foundations of mixed methods research. In A. Tashakkori & C. Teddlie (Eds.), SAGE handbook of mixed methods in social & behavioral research (2nd ed., pp. 95-118). Thousand Oaks, CA: Sage.

Diener, E., Emmons, R. A., Larsen, R. J., & Griffin, S. (1985). The Satisfaction with Life Scale. Journal of Personality Assessment, 49, 71-75.

Gross, J. J., & John, O. P. (2003). Individual differences in two emotion regulation processes: implications for affect, relationships, and well-being. *Journal of personality and social psychology*, *85*(2), 348.

Haynes, S. N., Mumma, G. H., & Pinson, C. (2009). Idiographic assessment: Conceptual and psychometric foundations of individualized behavioral assessment. *Clinical Psychology Review*, *29*(2), 179–191. https://doi.org/10.1016/j.cpr.2008.12.003

Karademas, E. C., Dimitraki, G., Papastefanakis, E., Ktistaki, G., Repa, A., Gergianaki, I., Bertsias, G., Sidiropoulos, P., Mastorodemos, V., & Simos, P. (2020). Emotion regulation contributes to the well-being of patients with autoimmune diseases through illness-related emotions: A prospective study. *Journal of Health Psychology*, *25*(13–14), 2096–2105. https://doi.org/10.1177/1359105318787010

Molenaar PCM. (2004) A manifesto on psychology as idiographic science: Bringing the person back into scientific psychology, this time forever. *Measurement.* 2:201–218

Mykletun, A., Stordal, E., & Dahl, A. A. (2001). Hospital Anxiety and Depression (HAD) scale: factor structure, item analyses and internal consistency in a large population. *The British journal of psychiatry*, *179*(6), 540-544.

Nazari, N., Sadeghi, M., Ghadampour, E., & Mirzaeefar, D. (2020). Transdiagnostic treatment of emotional disorders in people with multiple sclerosis: randomized controlled trial. *BMC psychology*, *8*(1), 1-11.

Onghena, P., Maes, B., & Heyvaert, M. (2019). Mixed Methods Single Case Research: State of the Art and Future Directions. *Journal of Mixed Methods Research*, *13*(4), 461–480. https://doi.org/10.1177/1558689818789530

Pavot, W. G., & Diener, E. (1993). Review of the Satisfaction with Life Scale. Psychological Assessment, 5, 164-172.

Pavot, W., & Diener, E. (2008). The satisfaction with life scale and the emerging construct of life satisfaction. *The journal of positive psychology*, *3*(2), 137-152.

Phillips, L. H., Saldias, A., McCarrey, A., Henry, J. D., Scott, C., Summers, F., & Whyte, M. (2009). Attentional lapses, emotional regulation and quality of life in multiple sclerosis. *British Journal of Clinical Psychology*, *48*(1), 101-106.

Sadeghi, Z., Ghoreishi, Z. S., Flowers, H., Mohammadkhani, P., Ashtari, F., & Noroozi, M. (2021). Depression, Anxiety, and Stress Relative to Swallowing Impairment in Persons with Multiple Sclerosis. *Dysphagia*, 1-8.

Saracutu, M., Edwards, D. J., Davies, H., & Rance, J. (2018). Protocol for a feasibility and acceptability study using a brief ACT-based intervention for people from Southwest Wales who live with persistent pain. *BMJ open*, *8*(11), e021866.

Sekhon, M., Cartwright, M., & Francis, J. J. (2017). Acceptability of healthcare interventions: an overview of reviews and development of a theoretical framework. *BMC health services research*, *17*(1), 1-13.

Methods of Analysis

Qualitative Data

The collection, analysis and final presentation of findings of the qualitative data will draw on guidelines of Interpretative Phenomenological Analysis (Smith et al., 2022). The analysis will be thorough and detailed, and every participant's interviews will be analysed separately in a case-by-case series. The Chief Investigator will first listen to the recording and read each transcript in order to gain familiarity with each case and make first observations, noting linguistic, descriptive, and conceptual codes throughout the transcripts. A longitudinal analysis will be conducted for each case conveying the diachronic developmental dimension in terms of their experience of the intervention and the impact on the experience and management of their Multiple Sclerosis. A case-by-case analysis and a careful and exhaustive examination across cases will be conducted to reveal the divergences and commonalities amongst the cases. The focus will be on the subjective experience of the MyMS-Ally psychological intervention and how participants appraise, perceive the intervention helpful or unhelpful, any change processes they might experience in relation to intervention processes. Analysis will be cyclical and iterative, constantly going back and forth in the data engaging in a dialogue. Emerging themes will be grouped in tables and diagrams and discussed in the research team, who will review all the steps of the analysis. Engaging in a more interpretative level of analysis, the meaning of each theme will be discussed as grounded on the participants' narratives. Following completion of the qualitative analysis, an experienced qualitative researcher, external to the research team, will audit the analysis and the final themes.

Quantitative Data

Descriptive statistics (means and standard deviations) will be calculated to provide insight into the participants' characteristics as well as indication of any potential changes in the mean scores between the different administration of the measures at four different points in time

(baseline, end of intervention, 1 month and 3 month follow up). Within subjects effects will also be calculated and reported. The final quantitative findings will be presented in a table reporting F, df, effect sizes, CI and p values.

References

Smith JA, Flowers P, Larkin M (2022) *Interpretative Phenomenological Analysis: Theory, Method, Research*. London: Sage (Second edition)

Risks, Burdens and Benefits

No serious adverse events are expected as a result of the study design and procedures. However, incidents might happen during the course of the study and the implementation of the psychological intervention:

- Participants might find the participation in a group intervention distressing and tiring.
- Participants might find the quantitative data collection process burdensome.
- Participants might not be able to attend the individual qualitative interviews.
- The group facilitator and the supervisor might make the decision that one or more participants cannot engage into the group intervention with the rest of the members.

The intervention and the study processes have been designed in a way that they respect the participants' needs and characteristics. The research team will follow the participants' pace and will take under consideration factors such as cognitive and physical fatigue in the intervention implementation and data collection processes. Given COVID restrictions, data collection processes will take place online. The processes will be discussed in the PPI involvement focus group.

Should any of the above adverse events occur, the participants have the right to withdraw from the study without having to justify their decision. If a participant withdraws, the CI will arrange for a check – in meeting with them if they accept the invitation and support resources will be provided to all participants in the Participant Information Sheet. Moreover, all data

from participants that withdraw from the study will not be used in the analysis and will be destroyed along with any personal details.

The CI, the independent researcher as well as the group facilitator will be assessing the risk for the participants. Should the risk becomes high for any of the participants in the course of the intervention implementation and the study, the CI will notify the health professional that referred the participant to the study. This is the only occurrence that confidentiality will be breached to ensure the participants' safety and wellbeing.

The intervention implementation schedule, the study processes and potential risks and benefits will be clearly outlined in the participant information sheet which will be given to all eligible patients with MS. Potential participants will also have a meeting with the CI in which these processes will be clarified and they will be able to ask questions. Participants will also be offered check – in meetings with the researcher after the end of collection of data and they will also have contact details of support sources in the participant information sheet.

The CI, the independent researcher and the group facilitator are not involved in the patients' regular care and there is no risk of changing the relationship with their healthcare professionals.

Benefits for participants

The benefits for participating in this study entail:

- It is aspired that participants will experience the benefits of the intervention in their experience and management of MS, their quality of life and emotion regulation.
- Taking part in a novel group psychological intervention which has been developed based on people with MS' accounts of needs and preferences, psychotherapy theory and consultation with practitioners.
- Contributing to ameliorating and finalising the intervention protocol which will be disseminated among people with MS and practitioners involved in their care.
- Participants' voices will be heard in terms of how they experience their MS and the psychological intervention through the qualitative processes of collection of data which will provide significant insights into the complexity of the disease and the effects of the intervention as well as future research and methodology in the field.

The research study and intervention have been developed based on a patient – centred approach and all procedures have been designed in a way that they protect the participants from the above adverse events and facilitate the benefits for them.

Risks for researchers

The chief investigator (CI) will be responsible for initial screening discussions with potential participants, design of the data collection, analysis of data, writing up of reports and dissemination. Qualitative interviews wil be conducted by an experienced independent researcher and the MyMS-Ally group psychological intervention will be facilitated by a trained Psychologist external to the research project.

The CI will have the support of the University of the West of England VC – ECR Award Research mentor with regards to the study processes. She will be able to discuss any issues that may occur and brainstorm on solutions and best practice. She will also have the support of the Bristol and Avon Multiple Sclerosis Centre team of health professionals. Data will be collectied online to minimise the risks related to COVID and every effort will be made to provide a safe environment for both participants and researchers.

The MyMS-Ally psychological group intervention facilitator will be recruited by the research team and will be trained on the MyMS-Ally group intervention before commencement of the group. They will have the supervision of a senior practitioner who will also be recruited for the purposes of the current study. The implementation of the group will most probably be online to minimise the risks related to COVID.

All measures will be taken to ensure that the study processes are safe for researchers, group facilitator and participants.
